# Autologous leukocyte-reduced platelet-rich plasma therapy for Achilles tendinopathy induced by collagenase in a rabbit model

**DOI:** 10.1038/srep19623

**Published:** 2016-01-19

**Authors:** Juan C. González, Catalina López, María E. Álvarez, Jorge E. Pérez, Jorge U. Carmona

**Affiliations:** 1 Grupo de Investigación Terapia Regenerativa, Departamento de Salud Animal, Universidad de Caldas, Calle 65 No 26-10, Manizales, Colombia

## Abstract

Leukocyte-reduced platelet-rich plasma (LR-PRP) is a therapy for tendinopathy of the Achilles tendon (TAT); however, there is scarce information regarding LR-PRP effects in rabbit models of TAT. We compared, at 4 and 12 weeks (w), the LR-PRP and placebo (PBS) effects on ultrasonography, histology and relative gene expression of collagen types I (*COL1A1*) and III (*COL3A1*) and vascular endothelial growth factor (VEGF) in 24 rabbits with TAT induced by collagenase. The rabbits (treated with both treatments) were euthanatised after either 4 or 12 w. A healthy group (HG (n = 6)) was included. At 4 and 12 w, the LR-PRP group had a no statistically different histology score to the HG. At w 4, the *COL1A1* expression was significantly higher in the LR-PRP group when compared to HG, and the expression of *COL3A1*from both LR-PRP and PBS-treated tendons was significantly higher when compared to the HG. At w 12, the expression of *COL3A1* remained significantly higher in the PBS group in comparison to the LR-PRP group and the HG. At w 4, the LR-PRP group presented a significantly higher expression of VEGF when compared to the PBS group and the HG. In conclusion, LR-PRP treatment showed regenerative properties in rabbits with TAT.

Tendinopathy of the Achilles tendon (TAT) can be classified as insertional and non-insertional tendinopathy, the latter representing the most common presentation of the disease^1^,^2^. The classical conservative treatments for non-insertional TAT include rest, eccentric exercises, shockwave therapy, non-steroidal anti-inflammatory drugs, and corticosteroids, among others. However, the two later drugs have been associated with deleterious effects on tendon healing^2^.

Platelet-rich plasma (PRP) is currently used for treatment of non-insertional (midbody) TAT. Recent publications suggest a beneficial effect of this substance in patients with TAT^3^,^4^. Rreports from several animal models of tendinopathy^5^,^6^, patients with naturally occurring TAT^7^,^8^ and *in vitro* studies^9^,^10^,^11^ have demonstrated beneficial effects (tissue anabolism and cell proliferation) of PRP on tendons.

PRP can be defined as plasma containing variable quantities of platelets and leukocytes in suspension^12^,^13^. According to the concentration of cellular components in PRPs, these substances can be classified into two groups: 1) leukocyte-reduced PRP (LR-PRPs (or pure PRP or leukocyte-poor PRP), which are preparations without leukocytes and with a low-density fibrin network after activation; and 2) leukocyte-concentrated PRP (LC-PRP) (leukocyte-PRPs) products, which are preparations with leukocytes and a low-density fibrin network after activation. When the PRP preparations are activated, they are transformed into a platelet rich gel (PRG). Therefore, the PRG from LR-PRP is known as LR-PRG, and the PRG from LC-PRP is known as LC-PRG^12^,^13^.

The rabbit model of TAT induced by collagenase has been employed for evaluating the effects of several drugs or procedures (either experimental or clinical) used for the treatment of this disease^14^,^15^,^16^,^17^,^18^. However, to the knowledge of the authors, no studies exist which evaluate the effect of PRP in this animal model of tendinopathy. Notably, this biological model has important translational usefulness, due to the anatomy of the Achilles tendon in rabbits and humans being quite similar^19^.

The anatomical condition of the rabbit’s Achilles tendon and scarce information regarding the effect of PRP in a rabbit model of TAT induced by collagenase were two important factor in proposing the study presented here. Furthermore, a LR-PRP was evaluated in this study, since *in vitro* evidence suggests that this PRP is more anabolic than leukocyte-concentrated PRP^9^,^10^.

The aims of the present study were to evaluate and compare, at weeks 4 and 12, the effects of LR-PRP and phosphate-buffered saline (PBS) on ultrasonographic (US) measurements, histology score, and relative gene expression of collagen type I (*COL1A1*), type III (*COL3A1*), and vascular endothelial growth factor (VEGF) in a rabbit model of TAT induced by collagenase.

It was hypothesised that tendons treated with LR-PRP would yield better US data and general histology score compared to tendons treated with PBS. It was also hypothesised that the parameters evaluated (including the relative gene expression) will be statistically similar between tendons treated with LR-PRP and tendons from the healthy control group.

## Results

The rabbits included in this study did not exhibit severe pain or discomfort during the experimentation period. All animals remained alive during the experimentation period. Tendon injury induced by collagenase treatment was diffuse, but primarily affected the central portion of the tendon. In general, the overall thickness of the tendon was affected by collagenase injection.

### Cell count and growth factor concentration in blood components

Cell count and growth factor concentration varied significantly (p < 0.05 _[t-paired test]_) between whole blood and LR-PRP, and between plasma and LR-PRG supernatants. LR-PRP presented a platelet enrichment of 128.5% and leukocyte reduction of 93.9% with respect to the basal cell counts in whole blood. Furthermore, LR-PRG supernatant presented a transforming growth factor beta 1 (TGF-β_1_) enrichment of 215.9% and a platelet-derived growth factor isoform BB (PDGF-BB) enrichment of 1266.2% with respect to the basal concentration of both GFs in plasma (Table 1).

### US findings

**Figure 1:**
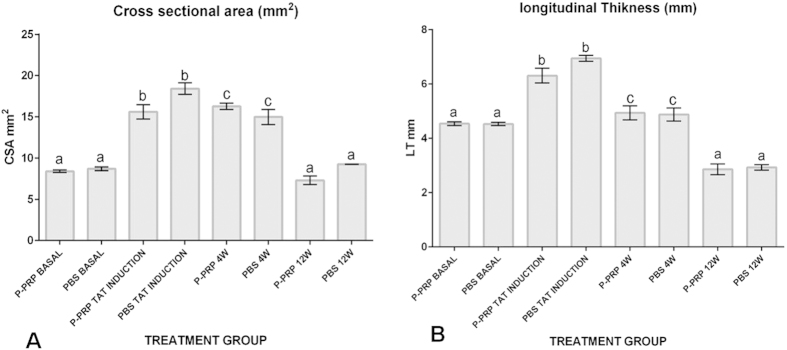
Means (± standard error of the means [s.e.m]) of the tendon ultrasonographic measures. (**A**). Tendon cross sectional area (CSA [mm^2^]). (**B**) Tendon longitudinal thickness (LT [mm]). W, week. (a–b) Lower-case letters denote significant differences by the Tukey test for the evaluated groups over time (*p < 0.05).

**Figure 2:**
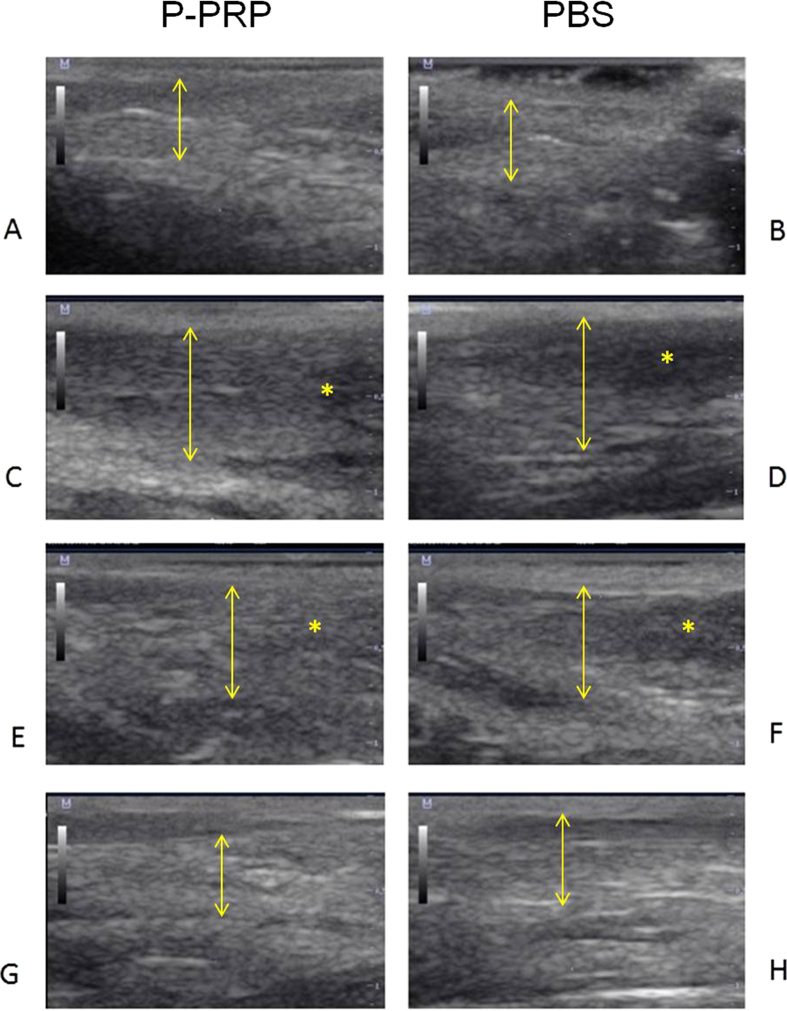
Tendon longitudinal ultrasonographic images. Normal basal tendon longitudinal ultrasonographic images in (**A**) LR-PRP- and (**B**) PBS-treated groups before collagenase tendinopathy induction. Tendon longitudinal ultrasonographic images in (**C**) LR-PRP- and (**D**) PBS-treated groups ten days after collagenase tendinopathy induction. Tendon longitudinal ultrasonographic images in (**E**) LR-PRP- and (**F**) PBS-treated groups 4 weeks after treatment. Tendon longitudinal ultrasonographic images in (**G**) LR-PRP- and (**H**) PBS-treated groups 12 weeks after treatment. ↕Tendon thickness indicator. Note that at any time no substantial differences are observed between both groups. However, there was an increase in the thickness of the tendons of both groups ten days after tendinopathy induction and 4 weeks after treatment. *Tendon damage indicator. Note hypoechoic zones in both groups ten days after tendinopathy induction and 4 weeks after treatment.

The tendon CSA (mm^2^) and tendon thickness (mm) presented a significant (p < 0.05 _[Tukey test]_) increase one week after TAT induction (Fig. 1A–B & Fig. 2A–H). The increase in both measurements remained for two additional weeks, then dramatically decreased until week 12 (Fig. 1A–B & Fig. 2A–H). At that point, both measurements were not significantly different to those tendon measures obtained prior to tendinopathy induction (Fig. 1A–B & Fig. 2A–H). No significant differences were observed for both US tendon measurements between the LR-PRP- and PBS-treated tendon groups throughout the duration of the experiment.

### Histology tendon injury scores

**Figure 3:**
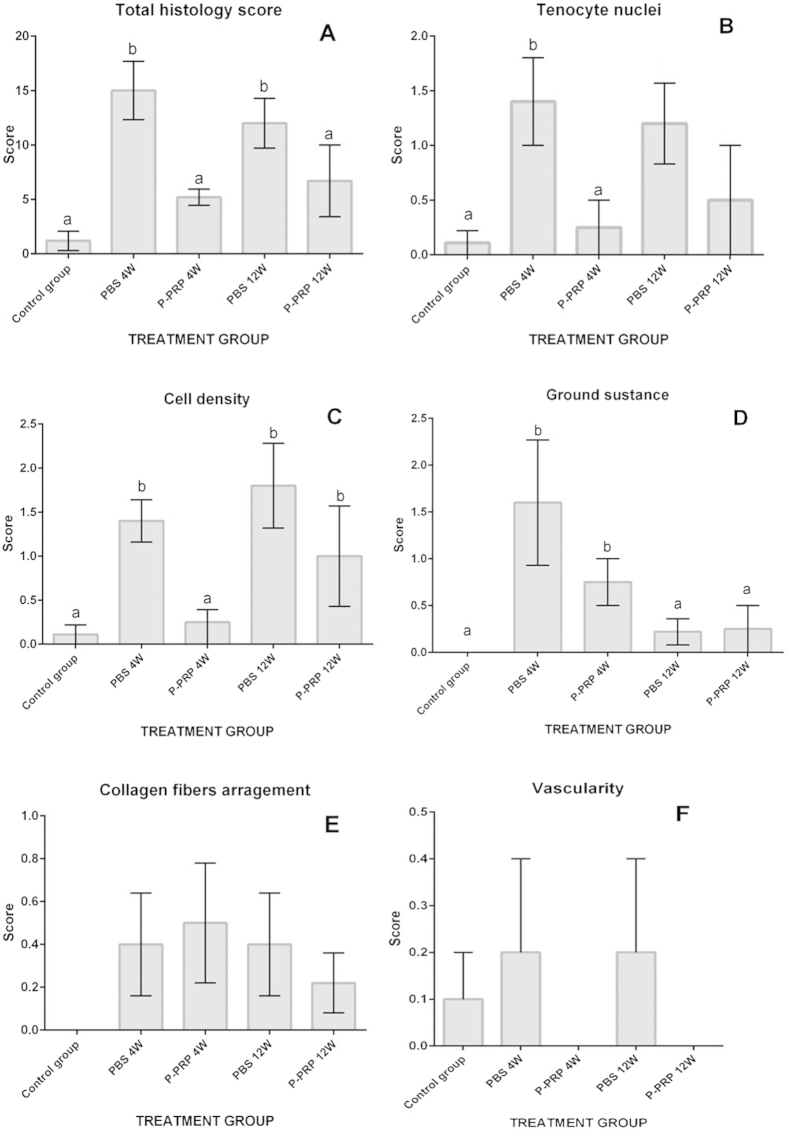
Means (± s.e.m) of the histology scores of tendon repair. (**A**) Total histology score. (**B**) Tenocyte nuclei score. (**C**) Cell density score. (**D**) Ground substance score. (**E**) Collagen fibers arrangement score. (**F**) Vascularity score. (a–b) Lower-case letters denote significant differences by the U-Mann-Whitney test for the evaluated groups over time (*p < 0.05).

At week 4, no significant differences were observed in the general histology score between the group of tendons treated with LR-PRP and the tendons of the healthy control group. In contrast, the total histology tendon injury score was significantly (p < 0.05 _[U-Mann-Whitney test]_) worse in tendons treated with PBS compared to the other two groups. At week 12, the total histology score was significantly different (p < 0.05 _[U-Mann-Whitney test]_) between all the evaluated groups (Fig. 3A). However, the group of tendons treated with LR-PRP exhibited a weak reduction in this score, when compared with the same group at 4 weeks.

Individual evaluation of the five parameters included in the histology score resulted in significant (p < 0.05 _[U-Mann-Whitney test]_) differences in only the roundness of the nuclei of the tenocytes (Fig. 3B), cell density (Fig. 3C), and ground substance deposition (Fig. 3D). Conversely, the collagen fibre arrangement (Fig. 3E) and vascularity histology scores (Fig. 3F) were not significantly different among all evaluated groups.

**Figure 4:**
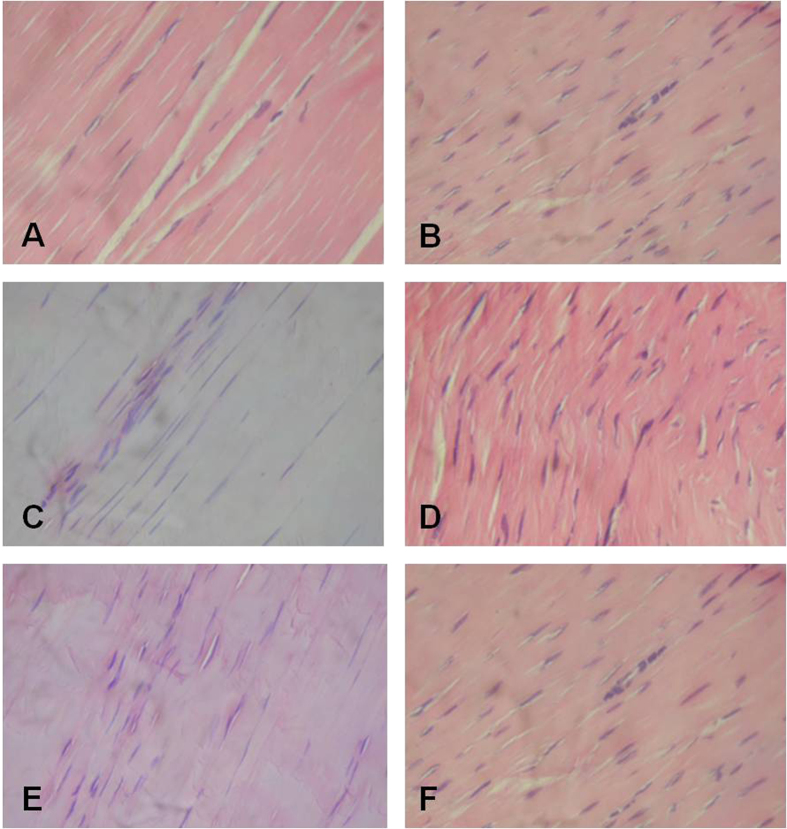
Tendon histology slides (H&E 40X). (**A**) Longitudinal section of a healthy Achilles tendon showing normal parallel orientation of the collagen fibres and presence of tenocytes with characteristic elongated nuclei. (**B**) Longitudinal section Achilles tendon 10 days after collagenase treatment showing obvious changes in the orientation of the collagen fibres, increased tenocyte number with roundness of their nuclei. (**C**) Longitudinal section of the Achilles tendon treated with LR-PRP after 4 weeks, showing a slight increase in the tenocyte number. However, these cells predominately maintain the typical elongated structure of their nuclei. (**D**) Longitudinal section of the Achilles tendon treated with PBS after four weeks, showing a slight change in the orientation of the collagen fibers; the tenocytes are increased and some of them exhibit elongate or rounded nuclei. (**E**) Longitudinal section of the Achilles LR-PRP-treated after 12 weeks, the quantity of tenocytes is diminished and the shape of their nuclei is elongated. (**F**) Longitudinal section of the PBS-treated Achilles tendon after 12 weeks in a normal orientation of its collagen fibres and showing a decreased number of tenocytes that conserve the roundness of their nuclei.

At week 4, the score for the roundness of nuclei in tenocytes was no significantly different between the tendons of the control group (Fig. 4A) and the tendons treated LR-PRP (Fig. 4C). However, the tendons treated with PBS presented a significantly worse score (p < 0.05 _[U-Mann-Whitney test]_) (Fig. 4D) than the aforementioned groups (Fig. 3B). At week 12, the score for the roundness of the nuclei of the tenocytes was not significantly different between LR-PRP and PBS groups (Figs 3B and 4E,F).

At week 4, scores for cell density were not significantly different for tendons treated with LR-PRP and the tendons from the healthy control group (Fig 4A,C and E), while significantly (p < 0.05 _[U-Mann-Whitney test]_) better than tendons treated with PBS at any time throughout the experiment (Fig. 4D,F). However, at week12, tendons treated with LR-PRP exhibited a significantly (p < 0.05 _[U-Mann-Whitney test]_) worse cell density score, which was not significantly different from scores observed for the same parameter in the tendons treated with PBS at any time (Figs 3C and 4E).

At week 4, tendons treated with LR-PRP or PBS presented significantly (p < 0.05 _[U-Mann-Whitney test]_) worse scores for ground substance compared to tendons from the healthy control group. At week 12, no difference was observed for this histological parameter in all evaluated groups (Fig. 3D).

### Gene expression results

#### COL1A1

**Figure 5:**
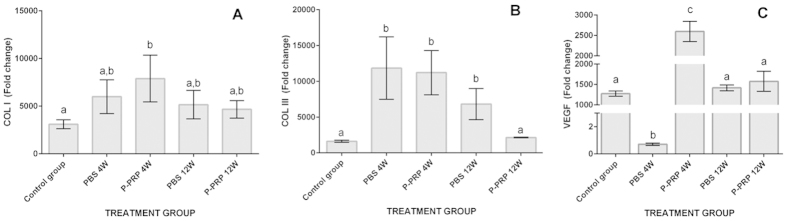
Means (± s.e.m) of the relative (fold change) gene expression. (**A**) Collagen type I relative expression. (**B**) Collagen type III relative expression. (**C**) Vascular endothelial growth factor (VEGF) relative expression. (a–b) Lower-case letters denote significant differences by the Tukey test for the evaluated groups over time (*p < 0.05).

At week4, the relative expression of *COL1A1* was significantly (p < 0.05 _[Tukey test]_) higher in tendons treated with LR-PRP when compared to tendons of the healthy control group (Fig. 5A). At week 12, no differences were observed for the expression of this gene in the evaluated groups (Fig. 5A).

#### COL3A1

At week 4, the relative expression of *COL3A1* from tendons treated either with LR-PRP or PBS was significantly (p < 0.05 _[Tukey test]_) higher when compared to the healthy control group (Fig. 5B). At week 12, the expression of *COL3A1* remained significantly (p < 0.05 _[Tukey test]_) higher in the PBS-treated group when compared to the LR-PRP and healthy control groups (Fig. 5B). However, no significant differences were observed for the relative expression of *COL3A1* between these two last groups.

#### VEGF

At week 4, the expression of VEGF was significantly (p < 0.05 _[Tukey test]_) lower in the tendons of the PBS group when compared to the expression of the gene in the tendons of the LR-PRP and healthy control groups (Fig. 5C). Conversely, tendons treated with LR-PRP presented a significantly higher expression of this GF when compared to the healthy control group (Fig. 5C). At week 12, VEGF expression was not significantly differentiated among all evaluated groups (Fig. 5C).

## Discussion

One goal of this study was to evaluate a LR-PRP preparation, because it up-regulates the expression of collagen type I and down-regulates the expression of catabolic cytokines and matrix metalloproteinases^9^,^10^, and previous *in vitro* studies have suggested that this kind of hemoderivate should be used clinically for tendinopathy treatment instead of LC-PRP^10^. In addition, a recent study in healthy rabbit tendons showed that LR-PRP preparations caused a significantly less acute inflammatory response at five days after injection when compared to LC-PRP preparations^20^.

The basal quantitative US measures, either CSA (mm^2^) or longitudinal thickness (mm) obtained in the study are in agreement with data from previous studies^19^,^21^. The ultrasonography was useful for monitoring the evolution of the tendinopathy in the rabbits of the study, but it was not useful for discriminating the effect of either LR-PRP or PBS at any time. Although some experimental studies in other animal species have demonstrated that US evaluation was useful for discriminating the effects between PRP from placebo groups^22^,^23^,^24^, this situation was not observed in the present study. On the contrary, our US findings were similar to those obtained in patients with TAT, which were treated with either PRP or saline, and no significant differences were noticed in the ultrasound evaluation at the end of the study^25^.

The histology findings showed the capacity of LR-PRP in ameliorating progressive tendon degeneration and promoting healing in this rabbit model of collagenase induced TAT. In the group of tendons treated with PBS, the histological score of tissue samples was always worse, which indicates that the collagenase injection unchained a progressive damage of the tendon (Fig. 4). This result is in line with previous studies using the same animal model^14^,^15^,^16^,^17^,^18^, in which the collagenase produced tendon damage characterised by loss of arrangement of collagen fibres, increased cell density and rounding of the nuclei of the tenocytes, amongst others^14^,^15^,^16^,^17^,^18^. In contrast, the group of tendons treated with LR-PRP showed a general histology score not statistically different to the tendons of the control healthy group (Fig. 3). Although not significantly different, it is noteworthy that the histology scores from tendons treated with LR-PRP were worse at 12 weeks than at 4 weeks. This scenario could indicate that several doses of LR-PRP may have been necessary for further improving the final histology outcome in this experiment. This finding could be useful for recommending two or more PRP doses in the treatment of patients with tendinopathies^26^.

Two pitfalls of the histology analysis performed in this study were that Masson’s trichrome stain was not used to evaluate collagen fibre arrangement^27^ and that immunohistochemical techniques were not used to detect vascularity by identifying CD31 positive cells in the tendon histology sections^28^. However, the tendons were evaluated by using a classic tendon histology score^29^,^30^, in which the tissue samples were stained with haematoxylin and eosin and alcian blue. This histology score has been previously used to evaluate a similar rabbit model of tendon injury^14^. Thus, we decided to use this histology score with its respective stains in order to obtain comparative data with other studies.

The expression of *COL1A1*was significantly elevated in tendons treated with LR-PRP at week 4, indicating that LR-PRP produces *COL1A1* up-regulation during early stage of the healing process, which restores the normal composition of inflamed tendons, which were affected by the action of bacterial collagenase. Similar findings have been reported in the transected Achilles tendons of rats treated with PRP^31^. On the other hand, the tendons treated with LR-PRP at 12 weeks presented a down-regulated relative *COL3A1* expression not statistically different to that of the control healthy group and the expression for this gene remained significantly increased in the tendons of the placebo group at any time. This finding demonstrated a regenerative effect of LR-PRP, because injured tendons with collagenase maintain an increased expression of this gene over time^32^. Furthermore, our results also corroborate the anabolic effects of leukocyte-reduced platelet concentrates on tendon explants, in which this substance down-regulated the expression of *COL3A1* in opposition to LC-PRP^9^,^10^.

The relative expression of VEGF was significantly lower in the group of the tendons treated with PBS at 4 weeks when compared to the control healthy group and the LR-PRP group. In contrast, the expression of this gene, at this same period of time, was significantly increased in the group of the tendons treated with LR-PRP in comparison to the healthy control and PBS groups. However, at week 12, the VEGF relative expression was not statistically different among the three groups. These findings corroborate that PRP injection produces an early up-regulation of VEGF that diminishes gradually over time^28^. It is known that the VEGF expression is critical for tendon healing, because this GF is scarcely expressed in the earliest phases of tendon injury^33^, thus the exogenous administration of PRP could be useful for improving tendon healing, as demonstrated in the present research.

An intriguing finding in this study was that there were not significant differences for the vascularity score among the groups evaluated, but the group of tendons treated with PRP presented a significant, and the highest expression of VEGF four weeks after treatment in comparison to the other two groups. There are few reports about the temporal and spatial expression of VEGF in healing tendons^34^,^35^ and its correlation with vessel formation^30^,^31^. Notably, Boyer *et al*.^35^, were not able to detect the specific type of cell (tenocyte or endothelial cells) that was expressing VEGF mRNA at the repair site of tendon lesions experimentally induced in dogs. They were unable to demonstrate that VEGF mRNA levels were correlated with revascularisation density at the injury zone. Currently, the angiogenic role of VEGF can only be demonstrated by selective inhibition of VEGF expression or production^30^,^31^. One explanation for the increased VEGF expression in the tendons treated with LR-PRP at four weeks was that the source of the mRNA of this GF could possibly come from cells contained or attracted by LR-PRP, like macrophages^36^, which disappeared once the healing process of the tendon was shifted to the remodelling phase^37^.

The present study presented some limitations. Firstly, although an animal model of TAT has served to evaluate the evolution of this disease and its response to either the LR-PRP or placebo treatment, the model does not reflect the conditions of naturally occurring TAT. Nevertheless, this is still a widely-accepted model of TAT, because lesions induced by collagenase mimic tendon injury and they are reproducible with a consistent histological lesion^14^,^15^,^16^,^17^,^18^. One additional limitation of this study was the short time period of experimental evaluation. However, this intended to develop an experimental protocol that included the mean time necessary for natural tendon repair following injury: three months^32^.

Other technical issues found in this study included biomechanical testing not being performed on analysed tendons, a control group evaluating the effect of plasma not being included, and ELISA human antibodies being used to detect rabbit growth factors. Biomechanical testing is a paramount procedure in orthopaedic research, due to its usefulness in demonstrating the mechanical properties of tissues, such tendons (tensile force and resistance). However, this procedure is technically complicated, as the structure of the tendon can limit its grip to the clamps of the testing device and consequently produce erroneous results. This situation is more critical when studying tissues of small animals such as rats or rabbits, which are commonly used in orthopaedic research^38^. This study had budget and ethical limitations in allotting independent groups for biomechanical testing, since the central portion of the tendons were divided into two equal portions for histological and gene expression analysis. However, the information obtained from histological and molecular analyses was useful for indicating the regenerative potential of LR-PRP in rabbit tendons.

The absence of a plasma-treatment group does not allow to establish if this substance could have regenerative properties similar to LR-PRP. However, as presented in Table 1, plasma preparations in this study presented a significantly lower concentration of growth factors evaluated in comparison to LR-PRP. Potentially, the tendon response to both treatments could be different. However, further studies are necessary to evaluate this hypothesis.

To the knowledge of the authors, no published studies exist evaluating the concentration of TGF-β_1_ and PDGF-BB in rabbit PRP with species-specific antibodies. However, both growth factors have been measured with human antibodies^39^,^40^,^41^, as in this study. Ideally, ELISA tests should be designed for specific rabbit samples, since human ELISAs can give positive signals, although it does not imply that these tests be accurate at all. However, this study used a human ELISA kits to determine rabbit TGF-β_1_ and PDGF-BB concentrations, because these growth factor are well-conserved between animal species^42^,^43^.

Overall, the results of this research demonstrate that LR-PRP produced regenerative effects in this rabbit model of TAT. The advantages of the PRP use in comparison with other cell therapies include the substance being autologous, and the technology for its processing is more simple and cost effective than other cell therapies potentially intended for tendinopathy treatment.

Histology data indicates that tendons treated with LR-PRP received better general score compared to tendons treated with PBS. Additionally, the relative gene expression of tendons treated with LR-PRP was not significantly different in comparison to tendons of the healthy control group. Furthermore, the results also suggest that additional LR-PRP doses could potentially prove useful for improving tendon healing. Further research is necessary to investigate this hypothesis.

## Methods

This study was approved by the ethical committee for animal experimentation of the Universidad de Caldas. Animals were fed and managed according to the guideless for animal experimentation.

### Animals

Thirty clinically healthy, skeletally mature, New Zealand rabbits with a body weight range of 3–4.2 kg were used. 24 rabbits were used to study the response to the tendon treatments (LR-PRP or PBS) and 6 animals were used as healthy controls.

### Study design

Rabbits (*n* = 24) were sedated with an intramuscular bolus of xilacyne 2% (5 mg/Kg). The region of both Achilles tendons was clipped and aseptically prepared. The centre of each tendon was injected with a 0.2 mL solution containing 250 UI of bacterial collagenase (*Clostridium histolliticum*) (Sigma-Aldrich, St Louis, MO, USA) by using an ultrasound-guide technique. Following tendinopathy induction, the analgesia of rabbits was maintained with tramadol chlorhydrate (10 mg/kg), *per os*, twice per day^44^, for five days.

**Figure 6:**
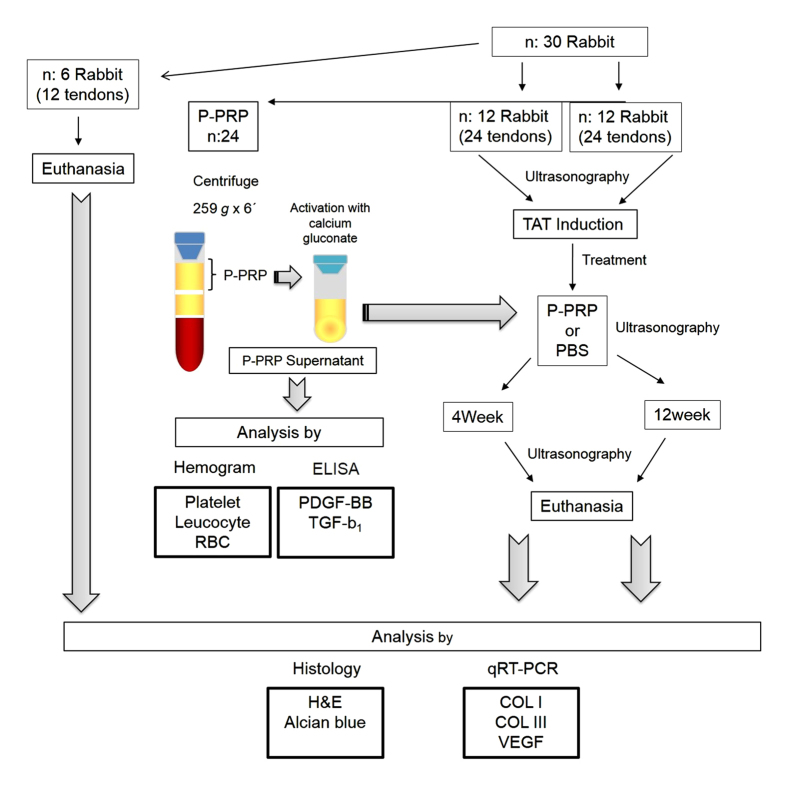
Schematic representation of the design of the study. H&E, haematoxylin and eosin; PBS, phosphate buffered saline; TAT, tendinopathy of Achilles tendon.

Ten days after TAT induction, the Achilles tendons of rabbits were randomly assigned to be treated with either autologous LR-PRP or PBS (Fig. 6).

Over the course of the experiment, the rabbits were clinically examined four times a day during the first three weeks, and then twice a day, to evaluate any complication associated with the induction of TAT. It was established that if some rabbits in the experiment could present a serious systemic or local complication, such as infection, tissue sloughing or severe pain, it should be euthanised.

### Euthanasia

The rabbits treated (with either LR-PRP or PBS) were randomly allotted into two euthanasia time groups. One group of 12 rabbits was euthanatised at week 4, while the other group of 12 animals was euthanatised at week 12 following the instauration of the treatments (10 days after TAT induction by collagenase injection). In addition, the clinically healthy (control) group (*n* = 6) was euthanatised at the end of the study to provide a histological and relative gene expression control.

The euthanasia of the animals was performed using an intravenous pentobarbital (30 mg/kg) injection prior to an intramuscular dose of xilacyne 2% (5 mg/kg). 50% of the central portion of the tendons were harvested and deposited in buffered formaldehyde for histology analysis. The other 50% of the sample was deposited in an RNA conserving solution (RNAlater, Life Technologies, Carlsbad, CA, USA) for quantitative analysis of gene expression of collagen type I (*COL1A1*), type III (*COL3A1*) and vascular endothelial growth factor (VEGF) by quantitative real-time PCR (qRT-PCR) (Fig. 6).

### Blood collection and autologous LR-PRP processing

Ten days after TAT induction, whole blood from the jugular vein of the rabbits was collected using a butterfly catheter No. 21G. Blood was deposited in two 4.5 mL 3.2% (v/v) sodium citrate tubes (BD Vacutainer, Becton Drive, Franklin Lakes, NJ, USA). Blood from one tube (4.45 mL) was used for haemogram and subsequent plasma processing through centrifugation at 1500 g for 15 min. The remaining tube was used for LR-PRP processing.

Briefly, the blood contained in the tube was centrifuged at 259 g for 6 min^45^. Then, the first top 50% plasma fraction was obtained and considered as LR-PRP (Fig. 6). Half of the total LR-PRP of every rabbit was used for haemogram and for later growth factor determination (after activation with calcium gluconate) and the other half for the experimental treatment. A total of 0.2 mL of either autologous activated LR-PRP (with calcium gluconate) or PBS was injected into the injured foci of the Achilles tendons (Fig. 6). LR-PRP was incubated with 10% calcium gluconate (10:1 ratio) at 37 °C for 6 h to induce its gelation (LR-PRG) and release of plasma supernatant rich in growth factors. This releasate was aliquoted and frozen at −80 °C for later determination of TGF-β_1_ and PDGF-BB.

### Growth factor determination

TGF-β_1_ and PDGF-BB concentrations were measured in plasma and LR-PRG supernatants from all rabbits after 6 h of incubation by using human enzyme-linked immunosorbant assay (ELISA) development kits (Human TGF-β1, DuoSet, DY240 and Human PDGF-BB DuoSet, DY220, R&D Systems, Mineapolis, MN, USA). Human GF antibodies were used, because TGF-β_1_ is a well-conserved protein in mammals^42^, and similar human ELISA kits have been successfully used for measuring both GFs in other rabbit PRP studies^40^,^46^. Standards provided for each ELISA kit were used for preparing each standard curve following the manufacturers’ instructions. Readings were performed at 450 nm.

### US evaluation

Previous to TAT induction, the tendons of the rabbits were evaluated for US measures to obtain the basal (normal) data of the Achilles tendons. All the measures were taken after a detailed clipping of the Achilles tendon zone. US evaluation was performed to determine the cross-sectional tendon area (CSA) (mm^2^) and (longitudinal) thickness (mm) at the level of the collagenase injury foci. The ultrasonographer was blinded to treatment group allocation. The measurements were taken from the central (injured) zone of the tendons in triplicate using an ultrasound device (Mindray DP 2200, Mindray Medical International Limited, China) with a probe of 7.5 MHz. US evaluation was performed prior to TAT induction (0 day), 7 days after TAT induction, and at 4 and 8 weeks after both treatments were injected.

### Histology evaluation

The tendon samples were dehydrated in serial alcohol concentrations, fixed in wax blocks, cut at 3 μm of thickness and stained with either haematoxylin and eosine (H&E) or alcian blue stains. The first stain was used to evaluate the roundness of the nuclei of the tenocytes, cell density, vascularisation, and the pattern of the collagen fibres. The alcian blue stain was used to determine the deposition of ground substance in the tendons^30^,^47^. All of the preparations were analysed using a light microscope in a 40x magnification by a modified Bonar´s semi-quantitative score (Table 2)^14^,^29^.

The five histological evaluated parameters were: (1) roundness of the nuclei of the tenocytes, (2) cell density, (3) ground substance, (4) collagen fibre arrangement, and (5) vascularity. These variables were quantified using a 0–3 scale, with 0 being normal and 3 being abnormal (Table 1). Thus, a totally normal tendon would score 0 and a maximally abnormal tendon would score 15. Three sections randomly selected from each sample were blindly evaluated and the averaged score was used for comparison^14^,^29^.

### Molecular evaluation

Tendon samples were pulverised in liquid nitrogen and mixed whit a TRIzol reagent (Life Technologies, Carlsbad, CA, USA) for 5 minutes. The samples were centrifuged for 10 minutes at 10000 g, and the supernatant was mixed with 20% chloroform (volume/volume) and then centrifuged for 15 minutes at 12000 g. The aqueous phases of the samples were removed and transferred to special columns (PureLink RNA Mini Kit, Life Technologies, Carlsbad, CA) for RNA extraction according to the manufacturer’s instructions. Then, the samples were assayed for quantitative gene expression levels in a qRT-PCR device (StepOnePlus Real-Time PCR System, Life Technologies, Carlsbad, CA, USA) using a SuperScript III platinum SYBR Green One-Step qRT-PCR kit (Life Technologies, Carlsbad, CA, USA). Primers for *COL1A1, COL3A1*, VEGF and GAPDH (glyceraldehyde 3-phosphate dehydrogenase) were designed and validated in the Colombian Center for Bioinformatics and Computational Biology (BIOS) (*COL1A1*: forward-CTCCGGCTCCTGCTCCTCTTAG, reverse-GTCCCTCGACTCCTGTGGTTTCC; *COL3A1*: forward-GCAGGGACTCCAGGTCTTAGAGG, reverse-CGTGTTCACCTCTCTCTCCCAGGG; VEGF: forward-CGTTAGCCTGGACTCTCTGGTGG, reverse-CTCCAACCCCTCTGTCTGTCTTCCTC and GAPDH: forward-GCCAAGGTCATCCACGACCAC, reverse-GCCATGCCAGTGAGTTTCCCG.

The relative change in gene expression was determined via the comparative 2^–ΔΔ*C*T^ method^48^, where ^Δ*C*T^ = CT, target - CT, GAPDH and ^Δ^^(Δ*C*T)^ = ^Δ*C*T^, stimulated - ^Δ*C*T^, control. GAPDH was used as the internal control (housekeeping gene), and tendon samples from the control healthy group were used as reference samples.

### Statistical analysis

Data were analysed using a statistical software (SPSS 19, IBM, Chicago, IL, USA). Values of every variable were plotted and analysed for normality using the Shapiro-Wilk test to determine the fit of data. All values of interest variables are presented as means (standard error of the means [s.e.m]). Data from hematologic parameters and growth factor concentration were evaluated by the t-paired test. Data from histology analysis were compared using a Kruskal-Wallis test followed, when necessary, by either the U-Mann-Whitney or Wilcoxon tests for *post-hoc* pairwise comparisons. Data from US, histology and molecular analyses were compared using a multivariate general linear model (GLM) followed, when necessary, by the Tukey test for *post-hoc* pairwise comparisons. A p < 0.05 value was accepted as statistically significant for all tests.

## Additional Information

**How to cite this article**: González, J. C. *et al*. Autologous leukocyte-reduced platelet-rich plasma therapy for Achilles tendinopathy induced by collagenase in a rabbit model. *Sci. Rep.* **6**, 19623; doi: 10.1038/srep19623 (2016).

## Figures and Tables

**Table 1:** Cell count and growth factor concentration in the blood components.

Variable	Whole blood	LR-PRP
Platelets × 10^3^/μ L	334.7 ± 16.2	430.4 ± 20.7*
Leukocytes × 10^3^/μ L	4.9 ± 0.4	0.3 ± 0.02*
RBCs × 10^6^//μ L	5.6 ± 0.08	0.02 ± 0.001*
	**Plasma**	**P-PRG supernatant**
TGF-β_1_ (pg/mL)	1076 ± 132	2324 ± 124*
PDGF-BB (pg/mL)	267.9 ± 45.8	3392 ± 238.7*

^*^Denotes statistically significant (*p < 0.05) differences between the variables in the same row by the paired t-test. PDGF-BB, platelet-derived growth factor BB; P-PRG, pure platelet-rich gel; LR-PRP, leukocyte reduced platelet-rich plasma; RBCs, red blood cells; TGF-β_1_, transforming growth factor beta 1.

**Table 2:** Bonar’s modified semi-quantitative score for tendon lesion determination.

Structure	Grade	1	2	3
	0			
Tenocytes	Inconspicuous elongated spindle shaped nuclei with no obvious cytoplasm at light microscopy.	Increased roundness: the ovoid nucleus becomes more round with clear cytoplasm.	Increased roundness and size; the nucleus is round, slightly enlarged and a small amount of cytoplasm is visible.	Nucleus is round, large with abundant cytoplasm and lacuna formation (chondroid change).
Cell density	Normal pattern	Slightly increase	Moderately increase	Severely increase
Ground substance (Alcian blue stain)	No stainable ground substance.	Stainable mucin between fibers but bundles still discrete.	Stainable mucin between fibers with loss of clear demarcation or bundles.	Abundant mucin through-out with inconspicuous collagen staining.
Collagen (with and without polarized light).	Collagen arranged in tightly cohesive well demarcated bundles with a smooth dense bright homogeneous polarization pattern with normal crimping.	Diminished fiber polarization; separation of individual fibers with maintenance of demarcated bundles.	Bundle changes; separation of fibers with loss of demarcation of bundles giving rise to expansion of the tissue overall and clear loss of normal polarization pattern.	Marked separation of fibers with complete loss of architecture.
Vascularity	Inconspicuous blood vessels coursing between bundles.	Occasional cluster of capillaries, less than 1 per 10 high power fields.	1-2 clusters of capillaries per 10 high power fields.	Greater than 2 clusters per 10 high power fields.
